# Change in prevalence of restrictive lung impairment in the U.S. population and associated risk factors: the National Health and Nutrition Examination Survey (NHANES) 1988–1994 and 2007–2010

**DOI:** 10.1186/s40248-015-0003-6

**Published:** 2015-02-28

**Authors:** Laura Kurth, Eva Hnizdo

**Affiliations:** Division of Respiratory Disease Studies, National Institute of Occupational Safety and Health/Centers for Disease Control and Prevention, 1095 Willowdale Road, Morgantown, WV 26505 USA

**Keywords:** FEV_1_/FVC, NHANES, Prevalence, Restrictive lung function, Spirometry

## Abstract

**Background:**

Data for the U.S adult population from the National Health and Nutrition Examination Survey (NHANES) were used to evaluate risk factors for a restrictive pattern on spirometry and estimate the change in its prevalence from the 1988–1994 to 2007–2010 sampling periods. Several previous epidemiologic studies used the Global Initiative for Chronic Obstructive Lung Disease fixed forced expiratory volume in 1 second (FEV_1_)/forced vital capacity (FVC) > 0.70 criteria for classifying restrictive pattern rather than the age-defined American Thoracic Society (ATS)/European Respiratory Society (ERS) lower limit of normal (LLN) criteria, which may lead to misclassification.

**Methods:**

Spirometry measurements from NHANES data for the 1988–1994 and 2007–2010 periods were analyzed to estimate the age-standardized prevalence of a restrictive pattern on spirometry and the change in prevalence over time for adults aged 20–79. A restrictive pattern was defined based on ATS/ERS LLN criteria as FEV_1_/FVC > LLN and FVC < LLN, and a moderate to more severe restrictive pattern was further evaluated using FEV_1_ < 70% predicted. The associations between demographic and other individual risk factors for restrictive lung impairment were examined using multivariable logistic regression models for the two consecutive time periods.

**Results:**

The overall age-standardized prevalence of restrictive pattern decreased significantly from 7.2% (1988–1994) to 5.4% (2007–2010) (p = 0.0013). The prevalence of moderate to more severe restrictive pattern also decreased significantly from 2.0% to 1.4% (p = 0.023). Factors positively associated with restrictive pattern on spirometry included age, female sex, white race, lower education, former and current smoking, and comorbidities including doctor-diagnosed cardiovascular disease, doctor-diagnosed diabetes, and abdominal obesity.

**Conclusions:**

The overall prevalence of restrictive pattern and moderate to more severe restrictive pattern decreased between the 1988–1994 and 2007–2010 survey periods despite a population increase in the proportion of comorbidities associated with restrictive pattern (i.e. diabetes and abdominal obesity). This suggests a decline in individual risk factors for restrictive pattern and a need for future research.

## Background

Restrictive pattern on spirometry is associated with functional limitations, fair or poor self-reported health status [1], reduced physical performance and physical impairment in older adults [2], and increased morbidity and mortality [3-6]. Restrictive pulmonary abnormalities are characterized by reduced lung compliance causing decreased total lung capacity. Restrictive lung impairment can be intrinsic (caused by diseases of the lung parenchyma) or extrinsic (caused by extraparenchymal diseases) [7-9]. Intrinsic restriction results from inflammation or scarring of lung tissue related to disorders, such as idiopathic pulmonary fibrosis, hypersensitivity pneumonitis, pneumoconiosis, or sarcoidosis [7]. Extrinsic restriction is linked to chest wall, pleura, or neuromuscular disorders affecting respiratory muscle function, such as scoliosis, kyphosis, pleural effusions, phrenic neuropathies, muscular dystrophy, and other nerve and muscle disorders [8,9].

The purpose of this study was to estimate the prevalence of a restrictive pattern on spirometry and the change in the prevalence over time for the U.S. population using two nationally-representative samples; National Health and Nutrition Examination Survey (NHANES) 1988–1994 and 2007–2010 data. We also estimate and compare the prevalence of demographic and other individual risk factors known to be associated with restrictive lung impairment and investigate their association with a restrictive pattern on spirometry for the two consecutive periods. National and global adult population-based epidemiologic studies report increased risk of a restrictive pattern on spirometry among older age groups [3,5,6], females [3,6], African American and other race/ethnicity groups [5], and smokers [3,5,6]. Epidemiologic studies also suggest that a restrictive pattern on spirometry is associated with a very low or very high body mass index (BMI) [3,5,6], and decreased lung volume is associated with excess adipose tissue around the central body region [10]. A restrictive pattern on spirometry is associated with increased rates of and mortality from stroke, hypertension, cardiovascular disease (CVD), and diabetes [3-5]. Exposure to substances found in the workplace including asbestos, silica, coal mine dust, and other organic and inorganic dusts are known to cause fibrotic tissue changes associated with restriction [11].

In clinical evaluation, a reduced total lung capacity (TLC) without evidence of expiratory flow reduction (increased or normal forced expiratory volume in the first second [FEV_1_]/vital capacity [VC] ratio) is interpreted as restriction [12]. However, TLC measurements are difficult to obtain for population-based studies, and epidemiologic studies often use spirometry volume measures to infer a restrictive pattern when TLC measurements are unavailable. Because NHANES spirometry testing does not include VC measures, forced vital capacity (FVC) was used in place of TLC and VC along with the lower limit of normal (LLN) to define a restrictive pattern on spirometry. LLN represents the lower 5^th^ percentile of the predicted value accounting for a person’s age, height, sex, and race/ethnicity. Several previous epidemiologic studies used the Global Initiative for Chronic Obstructive Lung Disease fixed-ratio criteria for classifying a restrictive pattern on spirometry including FVC < 80% predicted and FEV_1_/FVC ratio ≥ 0.7 [1-6,13]. Aging is associated with physiological lungs changes, including decreased chest wall compliance, respiratory muscle strength, and lung performance, affecting FEV_1_, FVC, and FEV_1_/FVC [14]. Using the fixed-ratio criteria rather than the ATS/ERS LLN criteria may inaccurately estimate the prevalence of restriction in older individuals [12,15].

## Methods

### Study population

The U.S. general population, 20–79 years of age, was studied using cross-sectional NHANES data from two time periods, 1988–1994 and 2007–2010. The National Center for Health Statistics (NCHS) conducts NHANES using a stratified, multistage, probability sampling design to survey a representative sample of the non-institutionalized, civilian U.S. population, and the NCHS Research Ethics Review Board approved the study protocol [16]. For both surveys, consent was obtained from participants and spirometry testing was performed in accordance with ATS recommendations using an Ohio 822/827 dry-rolling seal volume spirometer with biological filters (A-M Systems PFT Filter Kit B) [17]. The spirometry methods and equipment for the two periods differed by the following: 1) the flow-volume curve was displayed by the spirometry software for inspection by the technician in 1988–1994, while both the volume-time and flow-volume curves were available for inspection in 2007–2010; 2) the minimum number of acceptable maneuvers performed per test session was five (1988–1994) and three (2007–2010); 3) the test session repeatability requirement changed from 200 ml (1988–1994) to 150 ml (2007–2010); 4) the 2007–2010 survey included annual refresher training and bi-weekly, as opposed to monthly, quality control reports by the National Institute for Occupational Safety and Health [18,19].

During 1988–1994, 16,993 participants aged 20–79 were interviewed and, out of these, 15,331 (90% unweighted response rate) completed both the interview and exam survey components. In our NHANES 1988–1994 sample, 14,142 participants (92%) had at least two ATS acceptable FVC maneuvers, reliable spirometry tests [18], and a valid height measurement. During 2007–2010, 10,981 participants aged 20–79 completed the interview and exam components and were eligible for spirometry. Spirometry was not performed on some eligible participants due to limited exam time, subject refusal, or other reasons (n = 992) and for safety reasons (n = 817). NHANES 2007–2010 participants with invalid spirometry tests (n = 472) or a missing valid height measurement (2) were excluded from the analysis resulting in 8,698 participants (79%) in our sample [19].

### Definition of variables

The outcome variable, a restrictive pattern on spirometry was defined as FVC < LLN and FEV_1_/FVC > LLN. The severity of restrictive spirometry pattern was further evaluated using degree of FEV_1_ reduction per ATS/ERS criteria, and FEV_1_ < 70% of the predicted value indicated a moderate to more severe restrictive pattern [12]. Normative reference equations (developed from NHANES III data) accounting for age, height, sex, and race/ethnicity were used to determine the predicted and LLN FVC and FEV_1_ values for Whites, African Americans, and Mexican Americans [20]. Reference equations from NHANES III data were not available for Other Hispanic or Other race/ethnicity [20]. The reference equations for Mexican Americans were applied to Other Hispanics from the 2007–2010 survey [21]. The predicted and LLN FVC and FEV_1_ values for Other race/ethnicity were calculated by applying the correction factor for Asians (0.88) to the corresponding reference values for Whites [22]. Other Hispanic and Other race/ethnicity participants were included in the overall prevalence estimates, but separate results for these two groups are not reported [23].

Demographic covariates included in the analysis were age, sex, race/ethnicity (White, African American, Mexican American, or Other for NHANES 1988–1994 and White, African American, Mexican American, Other Hispanic, or Other for NHANES 2007–2010), and educational level. Risk factors known to be associated with restrictive impairment investigated in this analysis were: 1) doctor-diagnosed CVD (congestive heart failure, stroke, and heart attack), 2) doctor-diagnosed diabetes, 3) waist circumference (obtained by trained health technicians), 4) smoking status, and 5) occupational exposure [3-6,10,11]. A waist circumference ≥ 102 cm for males and ≥ 88 cm for females defined abdominal obesity based on 1998 National Institutes of Health obesity clinical guidelines [24]. Smoking status was derived from survey questions pertaining to smoking history, current smoking status, and age at initiation and cessation of smoking. Participants were categorized as never smokers (smoked < 100 cigarettes during their lifetime), former smokers, or current smokers. NHANES 2007–2010 participants reported on their past occupational exposure to mineral dusts, organic dusts, fumes, gases, or vapors in any job, as well as exposure to workplace tobacco smoke in their current job. Associations between a restrictive pattern and individual occupational exposures were not significant in the multivariable logistic regression model, so we combined the occupational exposure variables for the final analysis. Participants reporting exposure to dusts, fumes, gases, or vapors were considered to have “occupational exposure”.

### Data analysis

Statistical analyses were performed using SAS 9.3 (SAS Institute, Inc., Cary, NC). In the analyses, we took into account the sampling strategy (year and sampling unit) and used the NCHS individual sampling weights to obtain unbiased, nationally representative population size and survey prevalence estimates. The prevalence of restrictive pattern on spirometry (overall and by demographic factors) was calculated using SAS procedure Proc SurveyReg and age-standardized to the 2000 Census Population age structure [24]. Comparisons of prevalence between survey periods were performed with two-sided *t* tests using a Bonferroni corrected significance level to account for multiple comparisons (p < 0.05/number of implied comparisons). The associations between a restrictive pattern and demographic and other risk factors were evaluated using the multivariable logistic regression model. Separate models were fitted for the two NHANES survey periods. Using the NHANES 2007–2010 data, the association between self-reported occupational exposure and a restrictive pattern was also estimated. Interaction terms were evaluated in the model to investigate the combined effect of sex and individual risk factors (education, CVD, diabetes, waist circumference, and smoking status).

## Results

Between NHANES 1988–1994 and NHANES 2007–2010, the proportion of middle and older aged participants (ages 40–69) increased, and the proportion of younger aged participants (ages 20–39) decreased (Table 1). In NHANES 2007–2010, there were greater proportions of participants that were White, had at least some college education, those with diabetes, those with an obese waist circumference, and those that never smoked. Slightly higher mean BMI values in 2007–2010 suggest changes in weight-related anthropometric measurements of the survey population. However, in the more recent survey, there were slightly lower proportions of those with CVD and of current smokers. In NHANES 2007–2010, 54.9% (n *=* 4,636) reported occupational exposure.

**Table 1:** **Distribution of study participants**

	**NHANES 1988–1994**		**NHANES 2007–2010**	
	**Survey n** ^**a**^ **(%)**	**U.S. population n** ^**b**^	**Survey n** ^**a**^ **(%)**	**U.S. population n** ^**b**^
Total	14,142	1,615.7	8,698	1,715.0
*Age category, years*				
20–29	3,328 (23.5)	377.9	1,588 (18.3)	354.1
30–39	3,147 (22.3)	405.2	1,634 (18.8)	331.8
40–49	2,408 (17.0)	318.7	1,654 (19.0)	367.6
50–59	1,719 (12.2)	209.2	1,472 (16.9)	334.9
60–69	2,042 (14.4)	183.2	1,428 (16.4)	212.6
70–79	1,498 (10.6)	121.5	922 (10.6)	114.1
*Sex*				
Male	6,651 (47.0)	782.3	4,326 (49.7)	851.7
Female	7,491 (53.0)	833.5	4,372 (50.3)	863.4
*Race/ethnicity*				
White	5,486 (38.8)	1237.5	4,101 (47.2)	1,191.0
African American	4,095 (29.0)	173.0	1,633 (18.8)	182.9
Mexican American	4,002 (28.3)	82.7	1,607 (18.5)	147.2
Other Hispanic			960 (11.0)	86.3
Other	559 (4.0)	122.5	397 (4.6)	107.7
*Education*				
< High School	5**,**318 (37.8)	370.0	2,324 (26.8)	298.1
High School	4,488 (31.9)	548.7	2,066 (23.8)	405.8
Some college	2,409 (17.1)	345.0	2,471 (28.4)	524.2
College graduate	1,841 (13.1)	345.2	1,828 (21.0)	485.0
Cardiovascular disease	856 (6.1)	75.0	475 (5.5)	66.9
Diabetes	1,044 (7.4)	78.1	934 (10.8)	130.7
*Waist circumference*				
Non-obese	7,526 (54.8)	882.2	3,803 (43.7)	784.1
Obese	6,204 (45.2)	694.1	4,895 (56.3)	931.0
*Smoking status*				
Never smoker	6,844 (48.4)	728.8	4,621 (53.2)	928.3
Former smoker	3,350 (23.7)	410.0	2,028 (23.3)	403.6
Current smoker	3,947 (27.9)	476.8	2,046 (23.5)	382.8
Occupational exposure			4,636 (54.9)	
Workplace tobacco exposure			815 (15.2)	
*Mean height (cm) (SD)*	166.9 (9.7)		168.0 (10.1)	
*Mean weight (kg) (SD)*	76.1 (18.0)		82.5 (21.1)	
*Mean BMI (SD)*	27.3 (5.9)		29.2 (6.7)	

^a^NHANES participants aged 20–79 years. Sample excludes those with unreliable spirometry tests and missing values for standing height.

^b^Estimated weighted frequency of the U.S. population in 100,000.

The age-standardized prevalence of restrictive pattern declined significantly from 7.2% in 1988–1994 to 5.4% in 2007–2010 as did the prevalence of moderate to more severe restrictive pattern (2.0% to 1.4%) (p < 0.05) (Table 2). Although the decline in prevalence of restrictive pattern and moderate to more severe restrictive pattern was observed across all age categories, after Bonferroni adjustment it was only statistically significant among participants 50–59 years of age (p *=* 0.0001). The decline was also significant in females for restrictive pattern (p *=* 0.0004) and moderate to more severe restrictive pattern (p = 0.004). The prevalence of restrictive pattern was significantly lower in 2007–2010 for White participants (p *=* 0.0018), those with less than a high school education (p = 0.013), those with diabetes (p = 0.006), those with a non-obese waist circumference (p = 0.0002), and those that never smoked (p = 0.003) compared to 1988–1994. NHANES 2007–2010 participants with a non-obese waist circumference (p = 0.003) and that never smoked (p = 0.0001) had a significant decline in moderate to more severe restrictive pattern.

**Table 2:** **Weighted prevalence (%)** ^**a**^ **of restrictive pattern and moderate to more severe restrictive pattern**

	**Restrictive pattern (FVC < LLN, FEV**_**1**_ **/FVC > LLN)**			**Moderate to more severe restrictive pattern (FVC < LLN, FEV**_**1**_ **/FVC > LLN, FEV**_**1**_ **< 70%)**		
	**1988–1994**	**2007–2010**	**p** ^**b**^	**1988–1994**	**2007–2010**	**p** ^**b**^
Total^c^	7.2 (0.4)	5.4 (0.4)	0.0013^*^	2.0 (0.2)	1.4 (0.2)	0.023^*^
*Age category, years*						
20–29	5.2 (0.7)	3.5 (0.5)	0.06	0.4 (0.1)	0.4 (0.2)	0.50
30–39	5.2 (0.7)	4.2 (0.6)	0.17	0.7 (0.2)	0.4 (0.1)	0.15
40–49	5.7 (0.8)	4.9 (0.6)	0.23	1.2 (0.3)	0.8 (0.2)	0.16
50–59	11.6 (1.0)	7.0 (0.7)	0.0001^**^	3.5 (0.7)	2.2 (0.5)	0.07
60–69	11.2 (1.0)	9.0 (1.1)	0.07	5.1 (0.8)	4.3 (0.8)	0.25
70–79	7.9 (0.9)	6.9 (0.9)	0.23	4.6 (0.7)	2.8 (0.4)	0.029
*Sex* ^*c*^						
Male	7.4 (0.6)	6.3 (0.5)	0.10	1.7 (0.2)	1.3 (0.2)	0.09
Female	7.0 (0.5)	4.6 (0.4)	0.0004^**^	2.3 (0.2)	1.5 (0.2)	0.004^*^
*Race/ethnicity* ^*c*^						
White	7.5 (0.5)	5.4 (0.5)	0.0018^**^	2.0 (0.2)	1.4 (0.2)	0.019
African American	5.7 (0.4)	4.1 (0.6)	0.015	2.3 (0.3)	1.7 (0.4)	0.13
Mexican American	6.4 (0.4)	6.4 (0.7)	0.50	1.6 (0.3)	0.6 (0.2)	0.021
*Education* ^*c*^						
< High School	9.7 (0.9)	6.5 (0.7)	0.013^*^	3.1 (0.4)	2.1 (0.4)	0.07
High School	7.9 (0.7)	6.7 (0.8)	0.15	1.9 (0.3)	1.5 (0.4)	0.22
Some college	5.7 (0.5)	5.5 (0.7)	0.41	1.9 (0.4)	1.6 (0.3)	0.27
College graduate	5.1 (0.8)	3.8 (0.4)	0.07	1.1 (0.3)	0.8 (0.2)	0.20
*Cardiovascular disease* ^*c*^	11.5 (2.2)	9.7 (2.2)	0.30	4.4 (1.3)	4.0 (0.8)	0.41
*Diabetes* ^*c*^	14.7 (2.3)	8.2 (0.9)	0.006^*^	3.7 (1.0)	2.7 (0.5)	0.19
*Waist circumference* ^*c*^						
Non-obese	5.7 (0.5)	3.1 (0.3)	0.0002^**^	1.7 (0.2)	0.7 (0.1)	0.0003^**^
Obese	8.4 (0.7)	7.1 (0.5)	0.08	2.3 (0.3)	1.9 (0.3)	0.18
*Smoking status* ^*c*^						
Never smoker	6.7 (0.5)	4.8 (0.4)	0.003^*^	2.0 (0.2)	0.8 (0.1)	0.0001^**^
Former smoker	6.3 (0.8)	5.3 (0.6)	0.19	1.3 (0.3)	1.8 (0.3)	0.87
Current smoker	9.1 (0.8)	6.9 (0.8)	0.040	3.4 (0.5)	2.2 (0.4)	0.06

^a^Standard errors (SE) of the weighted prevalence estimates are given in parentheses.

^b^p from *t* test comparing prevalence between NHANES 1988–1994 and 2007–2010; p < 0.05 after Bonferroni corrected α for the number of comparisons at ^*^α = 0.05; ^**^α = 0.001.

^c^Age-standardized prevalence reported using the 2000 U.S. Census population.

Odds ratios (OR) and 95% confidence intervals (CI) for a restrictive pattern estimated from a multivariate analysis, for White, African American, and Mexican American participants, are presented in Table 3. There was an increasing trend in the odds ratios with age, which was more pronounced in the moderate to more severe category. The odds ratios for restrictive pattern were statistically significant for ages 50–59 and 60–69 in both surveys. Participants aged 40–79 (NHANES 1988–1994) and 50–79 (NHANES 2007–2010) had greater odds of having a moderate to more severe restrictive pattern than those 20–29 years of age. Males had lower odds of having a moderate to more severe restrictive pattern for both survey periods. In 1988–1994, African Americans and Mexican Americans were about 0.6 times as likely to have a restrictive pattern as Whites, and African Americans were also less likely to have a restrictive pattern (OR = 0.65, 95% CI 0.46–0.93). In NHANES 1988–1994, participants with at least some college education were significantly less likely to have a restrictive pattern compared to those with less than a high school education. However, in NHANES 2007–2010, a larger proportion of participants were college educated and educational attainment was no longer significantly associated with lower odds of restrictive pattern. Participants in both survey periods with CVD, diabetes, and an obese waist circumference were significantly more likely to have a restrictive pattern. The odds ratios for a moderate to more severe restrictive pattern were increased in both survey periods among those with CVD, an obese waist circumference, and current smokers. In NHANES 2007–2010, those with diabetes (3.66, 2.26–5.92) and former smokers (2.05, 1.22–3.44) were more likely to have a moderate to more severe restrictive pattern. Interaction variables between sex and individual risk factors were tested because males had systematically lower odds of moderate to more severe restrictive pattern, but consistent and statistically significant interaction terms were not found (p < 0.05).

**Table 3:** **Association between restrictive pattern and moderate to more severe restrictive pattern and risk factors**

	**Restrictive pattern (FVC < LLN, FEV**_**1**_ **/FVC > LLN)**		**Moderate to more severe restrictive pattern (FVC < LLN, FEV**_**1**_ **/FVC > LLN, FEV**_**1**_ **< 70%)**	
	**1988–1994**	**2007–2010**	**1988–1994**	**2007–2010**
	**OR (95% CI)** ^**a**^	**OR (95% CI)** ^**a**^	**OR (95% CI)** ^**a**^	**OR (95% CI)** ^**a**^
*Age category, years*				
20–29	1.00	1.00	1.00	1.00
30–39	1.18 (0.84–1.65)	1.10 (0.78–1.54)	1.90 (0.82–4.39)	1.03 (0.21–5.11)
40–49	1.13 (0.72–1.77)	1.11 (0.71–1.74)	2.93 (1.24–6.92)	2.27 (0.64–8.02)
50–59	2.15 (1.43–3.23)	1.57 (1.16–2.12)	7.42 (3.17–17.35)	5.00 (1.31–19.03)
60–69	1.90 (1.25–2.88)	1.76 (1.11–2.79)	9.85 (4.00–24.34)	7.54 (2.27–25.10)
70–79	1.11 (0.70–1.76)	1.29 (0.92–1.81)	8.57 (3.54–20.75)	3.99 (1.23–12.97)
*Sex*				
Female	1.00	1.00	1.00	1.00
Male	0.80 (0.61–1.06)	0.97 (0.70–1.36)	0.47 (0.34–0.67)	0.44 (0.27–0.72)
*Race/ethnicity*				
White	1.00	1.00	1.00	1.00
African American	0.62 (0.49–0.78)	0.65 (0.46–0.93)	0.90 (0.64–1.25)	0.88 (0.47–1.65)
Mexican American	0.66 (0.51–0.86)	1.29 (0.98–1.69)	0.64 (0.40–1.05)	0.35 (0.14–0.84)
*Education*				
< High school	1.00	1.00	1.00	1.00
High school	0.86 (0.64–1.14)	1.27 (0.90–1.80)	0.74 (0.40–1.36)	0.78 (0.37–1.64)
Some college	0.62 (0.42–0.90)	1.03 (0.71–1.48)	0.92 (0.50–1.69)	0.76 (0.38–1.50)
College graduate	0.52 (0.36–0.76)	0.77 (0.47–1.25)	0.53 (0.25–1.13)	0.51 (0.22–1.16)
*Cardiovascular disease*	1.58 (1.03–2.40)	1.51 (1.01–2.17)	1.85 (1.11–3.08)	2.24 (1.20–4.18)
*Diabetes*	1.60 (1.19–2.16)	2.65 (1.89–3.72)	1.48 (0.83–2.65)	3.66 (2.26–5.92)
*Waist circumference*				
Non-obese	1.00	1.00	1.00	1.00
Obese	1.83 (1.42–2.35)	2.14 (1.55–2.95)	2.43 (1.62–3.64)	3.20 (1.80–5.69)
*Smoking status*				
Never smoker	1.00	1.00	1.00	1.00
Former smoker	0.87 (0.62–1.21)	1.00 (0.74–1.36)	0.69 (0.44–1.08)	2.05 (1.22–3.44)
Current smoker	1.31 (1.04–1.65)	1.35 (0.98–1.86)	1.89 (1.24–2.86)	2.14 (1.24–3.68)
*Occupational exposure*				
No		1.00		1.00
Yes		1.05 (0.81–1.35)		1.09 (0.71–1.68)

White, African American, and Mexican American participants only.

^a^Adjusted for the other variables included in the table (age, sex, race/ethnicity, education, CVD, diabetes, waist circumference, smoking status, and occupational exposure for 2007–2010 population).

## Discussion

This study estimated the age-standardized prevalence of and risk factors associated with a restrictive pattern on spirometry in two population samples of U.S. adults surveyed approximately two decades apart. The overall prevalence of restrictive pattern significantly decreased from 7.2% in NHANES 1988–1994 to 5.4% in 2007–2010 (p = 0.0013). The decline in restrictive pattern was observed among those aged 50–59, females, Whites, those with less than a high school education, those with diabetes, those with a non-obese waist circumference, and never smokers (Table 2). The decline in moderate to more severe restrictive pattern was observed among those with a non-obese waist circumference and never smokers.

Participants aged 50–69 were significantly more likely to have a restrictive pattern compared to those 20–29 years of age (Table 3), which is consistent with prior studies [2,5,6]. Previous population-based studies suggest increased risk of restrictive pattern among females [3,6], which is consistent with our study for moderate to more severe restrictive pattern, specifically. An analysis of adults 25–74 years of age from NHANES 1971–1975 described an increased odds ratio of restrictive pattern (classified by the fixed-ratio criteria) among African Americans compared to Whites [5]. In contrast, our analysis of adults 20–79 years of age from NHANES 1988–1994 and 2007–2010 data described lower odds of ATS/ERS LLN defined restrictive pattern among African Americans compared to Whites.

Increased educational attainment served as a protective factor for restrictive pattern in NHANES 1988–1994. Participants with lower educational attainment are more likely to be obese [25], resulting in a higher likelihood of restrictive pattern, and are more likely to be employed in labor intensive jobs with elevated exposures to toxic substances [26]. Hazardous occupational exposures, especially fibrogenic types of mineral dust, may be related to increased restrictive pattern. However, occupational exposure was not significantly associated with a restrictive pattern in the NHANES 2007–2010 U.S. population sample; workplace exposure was not queried in NHANES 1988–1994. The effect of occupational exposure on a restrictive pattern was not modified by education in 2007–2010 when examining interaction variables for the combined effect of education and occupational exposure or duration of exposure (0–5 or ≥ 6 years). This lack of association may be due to the high proportion reporting occupational exposure (54.9%), non-specificity of the reported exposure, where occupational exposure ascertainment may not provide sufficient information on specific types of exposures known to cause restriction, including asbestos, silica, beryllium, and coal mine dust [11], and the healthy worker effect.

CVD, diabetes, and an obese waist circumference were associated with increased odds ratios for restrictive pattern, and various mechanisms have been postulated for these associations. Inflammatory mechanisms associated with CVD may lead to endothelial dysfunction and reduce both airflow in the lung and lung capacity. For example, inverse associations were described between reduced FVC and FEV_1_ and two markers of inflammation, serum C-reactive proteins [27] and fibrinogen [28]. Mechanisms for the association between restriction and diabetes include causative factors such as obesity, neuromuscular weakness, and vascular diseases [6]. An increased waist circumference due to excessive accumulation of subcutaneous and visceral fat around the central body region decreases lung volume by reducing diaphragm and thoracic cage mobility [10]. Abdominal obesity in adults is effectively measured by waist circumference [29], which is a better predictor of weight-related health problems in adults than BMI [30]. More detailed disease classification schemes including severity of CVD, diabetes, and obesity are necessary for future studies to better characterize the relationship between these comorbidities and a restrictive pattern on spirometry.

The prevalence of restrictive pattern was elevated in current smokers compared to former and never smokers for both survey periods. Smoking was significantly associated with increased odds of restrictive pattern in 1988–1994 and increased odds of moderate to more severe restrictive pattern in 1988–1994 and 2007–2010. Smoking can lead to irreversible interstitial lung changes, and smokers with interstitial lung abnormalities have reduced total lung capacity and increased risk of restrictive lung deficit compared to smokers without such abnormalities [31].

The decline in prevalence of restrictive pattern may be explained by changes in the proportion of risk factors associated with a restrictive pattern. The proportion with diabetes increased (Table 1), whereas the prevalence of restrictive pattern among those with diabetes, a non-obese waist circumference, and never smokers decreased (Table 2). The decline in prevalence of restrictive pattern in non-obese and never smokers may indicate a decline in the effect of diseases and the potential adverse effect of environmental and occupational exposures.

A study limitation of this paper is that differences in prevalence of restrictive pattern between survey periods may be affected by technical improvements in spirometry methodology. For example, the display of the volume-time curve for inspection by the technician in 2007–2010 may have improved some FVC measures and increased the prevalence with a normal FVC. The change in the test session repeatability requirement from 200 ml to 150 ml improved the precision and quality of spirometry tests in 2007–2010. The more lenient 1988–1994 standards may have led to false positive cases due to submaximal inspiratory efforts, air trapping, or a falsely low FVC. The changes in the spirometry technique are unlikely to have an effect on identifying significant predictors of restrictive spirometry pattern because the logistic regression analysis was conducted separately for each study period.

An additional limitation of the study is the reliability of a restrictive outcome inferred by spirometry. In clinical practice, measures of TLC are recommended in diagnosing restrictive lung disease [12,15]. Spirometry measures of FVC used in population-based epidemiologic studies tend to have low sensitivity and poor specificity for identifying restrictive patterns depending on the spirometry test quality [32]. However, lung volume tests are time-consuming, costly, and not easily adaptable to large population studies [3]. A decreased VC or FVC suggests restriction when FEV_1_/VC is normal or close to normal, but does not diagnose restriction, and is associated with reduced TLC approximately 50% of the time [12]. A decreased VC or a normal or slightly increased FEV_1_/VC ratio may be the result of submaximal inspiratory or expiratory efforts, peripheral airflow obstruction, air trapping, increased residual volume, and/or mild obstruction cases misclassified as restriction [12].

Spirometry data were not available for NHANES 1999–2006 survey years, but the 1988–1994 and 2007–2010 data provided a representative sample of adults. A z-score analysis of how well NHANES III reference equations fit the 2007–2010 data for healthy non-smokers indicated elevated observed FVC values for the 2007–2010 sample compared to those predicted with reference equations [33]. Furthermore, the higher observed FVC values result in lower mean FEV_1_/FVC values for NHANES 2007–2010 [33]. These results indicate that a new set of reference equations may be needed for the current U.S. population.

The current study classified restrictive pattern using ATS/ERS LLN criteria to account for the influence of a person’s age, height, sex, and race/ethnicity on lung volume measures [12]. Studies using NHANES spirometry data and classifying a restrictive spirometry pattern with a fixed-ratio criteria reported the prevalence of restrictive pattern on spirometry was 6.6% among participants 17 years of age and older (NHANES 1991–1994) [3], 7.6% among participants 20–79 years of age (NHANES 1988–1994), and 6.5% among participants 20–79 years of age (NHANES 2007–2010) [34]. Our results using the LLN reported a slightly lower prevalence of 7.2% among participants 20–79 years of age for NHANES 1988–1994 and 5.4% for NHANES 2007–2010. The discrepancy between the fixed-ratio criteria and LLN criteria prevalence estimates is likely due to misclassification of restrictive cases in older populations [14,15]. Assurance of uniform spirometry quality to establish valid measurements of FVC and comparability by age are also important aspects when comparing the prevalence of restriction across populations.

## Conclusions

In conclusion, the overall prevalence of restrictive pattern on spirometry and moderate to more severe restrictive pattern decreased from the NHANES 1988–1994 survey period to the 2007–2010 survey period. This study provides updated results regarding changes in the prevalence of restrictive patterns, using ATS/ERS LLN criteria to define a restrictive pattern on spirometry, and risk factors associated with a restrictive pattern. Additional evidence for the association between a restrictive spirometry pattern and age, CVD, diabetes, obesity, and smoking is provided. This study has public health implications by showing a decline in the prevalence of a restrictive pattern since 1988–1994 and by identifying restrictive lung impairment as a comorbidity of diseases including CVD and diabetes.

### Availability of Supporting Data

The data sets supporting the results of this article are available online to the public at http://www.cdc.gov/nchs/nhanes/nhanes_questionnaires.htm.

## Abbreviations

ATS: American Thoracic Society
BMI: Body mass index
CVD: Cardiovascular disease
CI: Confidence interval
ERS: European Respiratory Society
FEV_1_: Forced expiratory volume in 1 second
FVC: Forced vital capacity
LLN: Lower limit of normal
NCHS: National Center for Health Statistics
NHANES: National Health and Nutrition Examination Survey
OR: Odds ratio
TLC: Total lung capacity
VC: Vital capacity
